# Contact-Triggered Active Bonnet System for Pedestrian Head Injury Mitigation: Multibody Modelling and Experimental Validation

**DOI:** 10.3390/s26154743

**Published:** 2026-07-26

**Authors:** Alexandru Ionut Radu, Bogdan Adrian Tolea, Horia Beles, Florin Bogdan Scurt, Călin-Doru Iclodean

**Affiliations:** 1 Department of Automotive and Transport, Faculty of Mechanical Engineering, Transilvania University of Brașov, 500036 Brașov, Romania; alexandru.radu@unitbv.ro; 2 Department of Mechanical Engineering and Automotive, Faculty of Managerial and Technological Engineering, University of Oradea, 410087 Oradea, Romania; horia.beles@uoradea.ro (H.B.);; 3 Department of Automotive Engineering and Transports, Technical University of Cluj-Napoca, Muncii Bd. 103-105, 400114 Cluj-Napoca, Romania; calin.iclodean@auto.utcluj.ro

**Keywords:** active bonnet, pedestrian safety, contact sensing, multibody simulation, vehicle–pedestrian impact, head injury criterion, impact detection

## Abstract

**Highlights:**

**What are the main findings?**

- The proposed multibody framework showed satisfactory agreement with experimental pedestrian impact tests performed at 17 km/h and 29 km/h.
- The active bonnet system significantly reduced peak head acceleration and HIC15 values, with the effectiveness strongly influenced by pedestrian impact configuration and head trajectory.

**What are the implications of the main findings?**

- Active bonnet performance depends not only on vehicle speed, but also on pedestrian positioning and head impact location relative to the vehicle front-end geometry.
- The proposed contact-triggered multibody framework can support the development and preliminary optimization of integrated pedestrian protection systems and deployment strategies.

**Abstract:**

Pedestrian head injuries represent one of the most severe consequences of vehicle–pedestrian collisions, primarily resulting from the impact between the pedestrian head and the vehicle hood or windshield region. Active bonnet systems have been developed to mitigate injury severity by increasing the clearance between the hood and rigid engine components during impact; however, their effectiveness strongly depends on rapid impact detection and timely deployment. This study proposes an approach for analysing pedestrian impact dynamics and evaluating the design performance of a contact-triggered active bonnet system using MATLAB/Simulink and Simscape Multibody. The pedestrian–vehicle interaction is reproduced using spatial contact force models, while the bonnet deployment is initiated through a contact-based trigger mechanism coupled with a simplified actuator integrated into the hood hinge. The model was experimentally validated using controlled pedestrian impact tests at 17 km/h and 29 km/h, including frame-by-frame kinematic analysis and measured head acceleration signals. Additional simulations were performed at multiple vehicle velocities with and without bonnet deployment to evaluate the influence of impact configuration and pedestrian trajectory on head injury severity. The results indicate that the active bonnet system can reduce peak head acceleration and HIC15 values under several investigated impact conditions. The comparative simulations further show that the protective effect is influenced by pedestrian positioning and head trajectory relative to the vehicle front-end geometry, with higher effectiveness when the head impact remains within the deformable bonnet region.

## 1. Introduction

Pedestrian safety remains a major challenge in road traffic safety worldwide. Vulnerable road users, including pedestrians and cyclists, account for a significant proportion of traffic fatalities and severe injuries, particularly in urban environments where vehicle–pedestrian interactions frequently occur. Head injuries represent one of the most critical outcomes of pedestrian collisions, often leading to severe trauma or fatality due to the secondary impact between the pedestrian’s head and the vehicle hood or windshield area. Numerous accident reconstruction studies and injury analyses have highlighted that the head impact with the vehicle front structure is one of the primary determinants of injury severity in pedestrian crashes [1,2,3,4].

The dynamics of pedestrian impacts typically involve a sequence of events consisting of an initial contact between the vehicle bumper and the pedestrian’s lower extremities, followed by a rotation of the pedestrian body onto the hood surface and a secondary impact between the head and the upper vehicle structure. The severity of this secondary impact depends strongly on the vehicle geometry, impact velocity, and the stiffness characteristics of the hood and underlying components [5,6,7,8]. Previous research has demonstrated that vehicle front-end design parameters such as bumper height, hood leading edge, and hood stiffness significantly influence pedestrian kinematics and the resulting head injury risk [8].

To evaluate pedestrian head injury severity, the Head Injury Criterion (HIC) has been widely adopted as a biomechanical injury metric. The HIC value is derived from the resultant head acceleration during impact and provides an estimation of the likelihood of severe head trauma [9,10,11]. Consequently, reducing head acceleration during vehicle–pedestrian collisions has become a primary objective in modern pedestrian protection strategies.

In recent years, automotive manufacturers and regulatory organizations have introduced several passive and active safety systems aimed at reducing pedestrian injury severity. Passive countermeasures include energy-absorbing hood structures, deformable bumpers, and optimized vehicle front-end geometries designed to reduce injury risk during impact [12,13,14]. In addition to passive measures, active pedestrian protection systems have been developed to mitigate the consequences of head impacts by modifying the vehicle structure during the collision event.

One of the most widely investigated active protection technologies is the active bonnet (or deployable hood) system, which increases the clearance between the hood surface and the rigid engine components immediately after impact detection. By lifting the rear part of the hood, the system creates additional deformation space that allows the hood to absorb a larger portion of the impact energy, thereby reducing head acceleration and injury severity [15,16,17,18]. Experimental investigations have demonstrated that deployable hood systems can significantly decrease the Head Injury Criterion in head form impact tests compared to conventional hood structures [15].

The effectiveness of active bonnet systems depends strongly on the ability to rapidly detect the pedestrian impact and trigger the deployment mechanism within a very short time interval. Various sensing technologies have been proposed for this purpose, including pressure sensors embedded in the bumper, acceleration sensors, and contact-based detection mechanisms capable of identifying the initial impact between the vehicle and the pedestrian’s lower limb [19,20,21]. These sensing systems must reliably distinguish pedestrian impacts from other events such as collisions with road infrastructure or small obstacles, while ensuring sufficiently fast response times to allow the hood deployment before the head impact occurs.

In parallel with experimental research, computational modelling has become an essential tool for investigating pedestrian impact dynamics and evaluating pedestrian protection systems. Multibody simulation methods are widely used for studying vehicle–pedestrian interactions due to their computational efficiency and ability to reproduce the global kinematics of pedestrian motion during impact events [22,23,24]. These models enable researchers to analyse various design configurations and safety technologies without the need for extensive physical testing.

Several studies have employed multibody simulation frameworks to analyse pedestrian kinematics and evaluate the effectiveness of different vehicle front-end designs and active safety systems [7,8,24]. However, reliable simulation results require proper validation using experimental data obtained from controlled impact tests. Model validation is therefore an essential step in ensuring that the simulated head acceleration responses and injury metrics accurately represent real-world impact conditions [25,26,27].

Previous studies have also reported the injury reduction potential of integrated pedestrian protection systems and autonomous emergency braking strategies [28,29,30].

The objective of the present study is to develop and validate a multibody simulation framework for analysing pedestrian impact dynamics and evaluating the effectiveness of a sensor-triggered active bonnet protection system. The vehicle–pedestrian interaction is modelled using MATLAB/Simulink and Simscape Multibody (R2024b, The MathWorks, Inc., Natick, MA, USA), where spatial contact force models are used to reproduce the interaction between the pedestrian body segments and the vehicle front structure. A contact-based sensing mechanism is implemented to detect the initial interaction between the pedestrian and the vehicle, providing the trigger signal required for bonnet deployment.

To ensure the reliability of the simulation model, the numerical results are validated using experimental data obtained from a controlled pedestrian impact test. The simulated head acceleration responses are compared with the measured acceleration signals in order to evaluate the accuracy of the model. Following the validation stage, the model is employed to investigate the influence of an active bonnet system on pedestrian head injury severity by comparing impact scenarios with and without bonnet deployment.

The proposed approach provides a computational framework that integrates impact sensing, multibody modelling, and injury assessment, enabling the evaluation of active pedestrian protection systems aimed at reducing head injury risk in vehicle–pedestrian collisions.

Unlike pressure-based systems embedded in the bumper, accelerometer-based detection strategies, or predictive approaches based on vehicle dynamics, the proposed method employs a direct contact-triggered sensing mechanism integrated within the multibody simulation framework. This allows a transparent and computationally efficient evaluation of the interaction between sensing, actuation timing, and pedestrian kinematics.

Furthermore, the novelty of this work lies in the integration of contact-based detection, multibody modelling, actuator dynamics, and experimental validation within a unified MATLAB/Simulink/Simscape environment, enabling a consistent assessment of pedestrian injury mitigation strategies.

The main contributions of this study can be summarized as follows:

1. Proposing a contact-triggered active bonnet evaluation approach focused on pedestrian head impact mitigation;
2. Analysing the influence of pedestrian impact configuration, head trajectory, and bonnet deployment timing on head injury indicators;
3. Experimentally validating the numerical approach using controlled pedestrian impact tests at 17 km/h and 29 km/h;
4. Assessing the influence of actuator delay and torque on active bonnet performance.

## 2. Multibody Modelling and Sensor-Based Impact Detection

### 2.1. Governing Equations

To provide a theoretical description of the implemented simulation framework, the main governing equations used in the multibody vehicle–pedestrian model are summarized in this section. Since the complete model includes multiple interconnected rigid bodies, several contact interfaces, and an active bonnet actuation mechanism, the analytical description is presented in compact form, focusing on the equations that govern the overall dynamics, contact response, bonnet actuation, and injury assessment. The vehicle–pedestrian system can be described using the general equation of motion of a constrained multibody system:

(1) $$M(q)\;\ddot{q}+C(q,\dot{q})+G(q)=Q_{ext}+Q_{c}$$

where *q* is the vector of generalized coordinates, *M*(*q*) is the mass matrix, *C*(*q*,$\dot{q})$ contains inertial and coupling terms, *G*(*q*) represents gravitational effects, *Q_ext_* denotes the externally applied generalized forces and torques, and *Q_c_* represents the generalized contact forces. In the present case, external actions include the prescribed vehicle motion and the bonnet actuator torque, while the contact forces arise from interactions between the pedestrian and the vehicle, as well as between the pedestrian and the ground.

The contact interactions were modelled using a smooth spring–damper formulation. The normal contact force is expressed as:

(2) $$F_{n}=\left\{\begin{array}{cc} k\;\delta +c\;\dot{\delta }, & \delta >0\\ 0, & \delta \leq 0 \end{array}\right.$$

where *F_n_* is the normal contact force, *k* is the contact stiffness, *c* is the damping coefficient, *δ* is the penetration depth, and $\dot{\delta }$ is the relative penetration velocity. This formulation enables continuous force generation when contact occurs and avoids numerical discontinuities associated with rigid impact formulations. Different values of *k* and *c* were assigned to the lower limbs–bumper, torso–hood, head–hood, and body–ground contact pairs, as presented in the corresponding model parameter tables.

Tangential interaction was introduced through a simplified friction law, written in the form:

(3) $$|F_{t}|\;\leq \;\mu F_{n}$$

where *F_t_* is the tangential contact force, and *μ* is the friction coefficient. In the implemented model, a constant friction coefficient was assumed for all contact pairs, providing a simplified but consistent representation of sliding effects during impact.

The dynamic behaviour of the active bonnet was modelled by considering the hood as a rigid body rotating about a revolute joint located at the hinge. Its rotational dynamics can be expressed as:

(4) $$J_{h}\;\ddot{\theta }+c_{h}\;\dot{\theta }+k_{h}(\theta -\theta_{0})=T_{act}(t)$$

where *J_h_* is the equivalent rotational inertia of the bonnet relative to the hinge, *θ* is the bonnet angular displacement, *θ*_0_ is the equilibrium angular position, *k_h_* is the hinge rotational stiffness, *c_h_* is the hinge damping coefficient, and *T_act_*(*t*) is the actuator torque applied to the bonnet joint. This equation reflects the balance between inertial effects, elastic restoring torque, damping resistance, and the external actuation torque responsible for bonnet deployment.

The actuator torque is governed by the contact-based trigger logic. In simplified form, the activation law can be written as:

(5) $$T_{act}(t)=\left\{\begin{array}{cc} T_{0}, & s_{c}(t-t_{d})>0\\ 0, & s_{c}(t-t_{d})\leq 0 \end{array}\right.$$

where *T*_0_ is the prescribed actuator torque, *s_c_*(*t*) is the contact signal generated by the impact detection mechanism, and *t_d_* is the deployment delay. This formulation reproduces the logic used in the Simulink implementation, where the bonnet is activated only after contact detection and deactivated once the corresponding condition is no longer satisfied.

In this formulation, the deployment delay is represented as an equivalent activation delay applied to the trigger signal. The detailed actuator rise curve is not explicitly modelled; therefore, *t_d_* should be interpreted as an equivalent response parameter rather than as a full physical actuator model.

To evaluate the dynamic severity of the impact, the resultant head acceleration was computed from its three orthogonal components as:

(6) $$a_{res}(t)=\sqrt{a_{x}^{2}(t)+a_{y}^{2}(t)+a_{z}^{2}(t)}$$

where *a_x_*(*t*), *a_y_*(*t*), and *a_z_*(*t*) are the head acceleration components measured or simulated along the three axes of the local coordinate system. This resultant acceleration was used both for comparison with experimental measurements and for the calculation of head injury criteria.

The severity of the head impact was quantified using the Head Injury Criterion (HIC), defined as:

(7) $$HIC=\underset{t_{1},t_{2}}{max}\left[(t_{2}-t_{1}){\left(\frac{1}{t_{2}-t_{1}}\int_{t_{1}}^{t_{2}}a(t)\;dt\right)}^{2.5}\right]$$

where *a*(*t*) is the resultant head acceleration expressed in units of gravitational acceleration *g*, and [*t*_1_, *t*_2_] is the time interval over which the criterion is maximized. In this study, the HIC15 formulation was adopted, meaning that the duration of the interval satisfies:

(8) $$t_{2}-t_{1}\leq 15\;\mathrm{ms}$$

This criterion accounts for both the amplitude and duration of head acceleration and is widely used in automotive safety analysis to assess head injury risk.

Since the numerical implementation was performed using a fixed-step simulation scheme, the HIC computation in post-processing was based on discretized acceleration data. For a sampled acceleration signal, the average acceleration over a candidate interval can be approximated as:

(9) $${\bar{a}}_{ij}=\frac{1}{t_{j}-t_{i}}\int_{t_{i}}^{t_{j}}a(t)\;dt$$

and the corresponding discrete HIC value becomes:

(10) $$HIC_{ij}=(t_{j}-t_{i})\;{\bar{a}}_{ij}^{\;2.5}$$

The final HIC15 value is obtained by searching for all admissible intervals satisfying Equation (10) and selecting the maximum value. This discrete implementation ensures consistency between the simulated signals and the sampled experimental data used in the validation stage.

### 2.2. Multibody Model of the Vehicle–Pedestrian System

**Figure 1:**
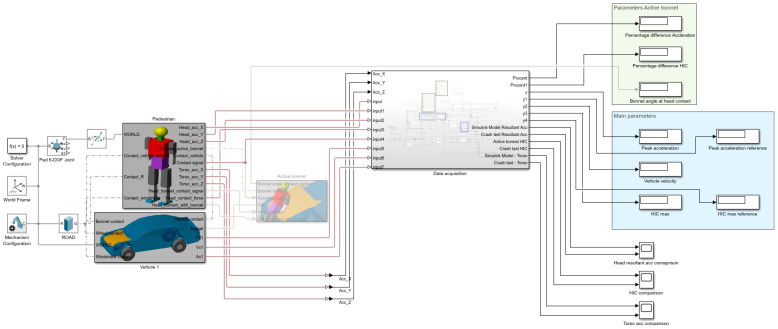
Simulink/Simscape implementation of the multibody vehicle–pedestrian system, including the pedestrian model, vehicle front-end, active bonnet mechanism, contact interactions, and data acquisition blocks.

The vehicle–pedestrian impact scenario was modelled using a multibody simulation approach implemented in MATLAB/Simulink with the Simscape Multibody environment. Multibody modelling is widely used for analysing pedestrian impacts due to its ability to reproduce the global kinematics of the human body while maintaining relatively low computational cost compared to finite element models. The multibody model of the vehicle–pedestrian system was developed in the Simulink/Simscape environment, integrating the pedestrian model, the vehicle front-end structure, and the active bonnet mechanism into a unified simulation framework. The overall architecture of the implemented model is illustrated in Figure 1.

As shown in Figure 1, the model consists of a rigid multibody representation of the pedestrian, coupled with a simplified vehicle front structure and a sensor-triggered active bonnet system. The interaction between the pedestrian and the vehicle is governed by contact elements, while the resulting kinematic and dynamic responses are monitored through dedicated data acquisition blocks for subsequent injury assessment.

**Figure 2:**
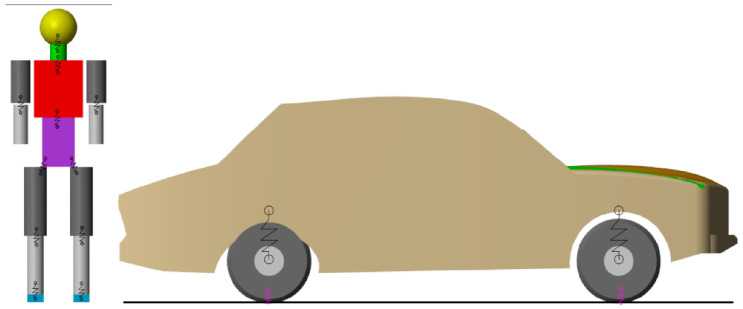
Simplified multibody representation of the pedestrian and vehicle front-end geometry, highlighting the articulated pedestrian segments and the contact interface with the vehicle hood. The colours indicate the head, torso, pelvis, upper limbs, and lower limbs of the simplified pedestrian model.

To provide a clearer understanding of the physical representation of the modelled system, a simplified geometric view of the pedestrian and vehicle multibody configuration is presented in Figure 2.

As illustrated in Figure 2, the pedestrian is modelled as a multibody system composed of rigid segments connected primarily through spherical joints, allowing three rotational degrees of freedom. Selected joints, such as the knees and the head–neck connection, are modelled using revolute joints to constrain the motion to a single dominant rotation axis.

Since the pedestrian impact kinematics are strongly influenced by the vehicle front-end geometry, several representative geometric parameters of the simplified vehicle model are summarized in Table 1.

The presented geometric characteristics influence the pedestrian lower-limb contact point, torso rotation mechanism, and subsequent head trajectory during impact. In particular, the leading-edge bonnet height and windshield angle play an important role in determining whether the head impact occurs primarily within the deformable bonnet region or migrates toward the windshield area at higher impact velocities.

This hybrid joint modelling approach was adopted to balance biomechanical realism and numerical stability.

The proposed model was primarily calibrated and validated for head impact dynamics and HIC-based injury assessment. Consequently, the framework should not currently be considered suitable for detailed thoracic, pelvic, or lower-limb injury prediction.

### 2.3. Active Bonnet System

The active bonnet system was implemented as a sensor-triggered mechanism designed to reduce the severity of head injuries during pedestrian–vehicle impact. The system is based on a torque-driven actuation applied at the bonnet hinge, allowing rapid upward rotation of the bonnet immediately after contact detection.

**Figure 3:**
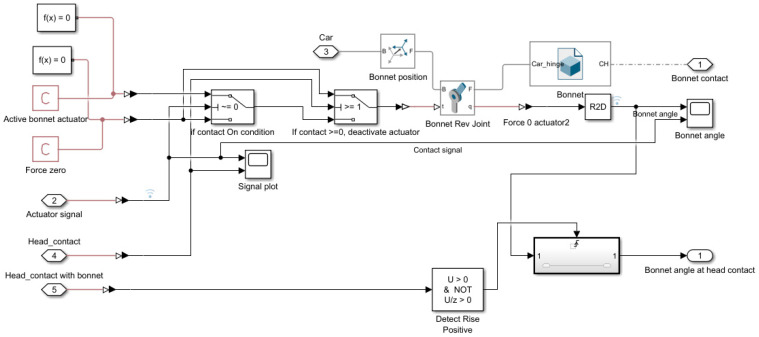
Simulink implementation of the active bonnet control system, including the contact detection logic, actuator activation, and bonnet rotation through a revolute joint.

The actuator was modelled as an equivalent constant-torque source with fixed deployment delay. This simplified representation does not aim to reproduce the detailed behaviour of pyrotechnic or electromechanical actuators, but rather to capture the first-order dynamics of bonnet deployment in a numerically stable and controllable manner, suitable for comparative analysis. The constant-torque actuator was adopted as a simplified equivalent representation of the deployment mechanism. The purpose of this approximation was not to reproduce the detailed nonlinear force–time curve of a pyrotechnic or electromechanical actuator, but to evaluate the influence of bonnet lift timing and angular displacement on pedestrian head impact response. Therefore, the actuator parameters should be interpreted as equivalent modelling parameters used for comparative analysis. A detailed actuator model including rise time, saturation, locking behaviour, and nonlinear deployment characteristics will be considered in future work. The control logic and actuation scheme of the system are illustrated in Figure 3.

As shown in Figure 3, the bonnet is modelled as a rigid body connected to the vehicle structure through a revolute joint, representing the physical hinge mechanism. The actuation is achieved by applying a torque input to this joint, simulating the deployment of an active bonnet system.

The activation of the actuator is governed by a contact-based signal derived from the interaction between the pedestrian and the vehicle front-end. When the contact condition is detected, the actuator is triggered, applying a predefined torque to the hinge joint. This results in a rapid increase in the bonnet angle, creating additional deformation space between the hood and the underlying rigid components. To ensure a realistic behaviour, the actuator is controlled using a conditional logic structure. The torque is applied only during the contact phase, while a zero-force condition is imposed when the contact signal is inactive. This approach prevents continuous actuation and allows the bonnet to return toward its equilibrium position after the impact event. The rotational behaviour of the bonnet is further influenced by the mechanical properties assigned to the hinge joint, including stiffness and damping. These parameters were introduced to replicate the resistance and energy dissipation characteristics of a real bonnet mechanism. The resulting bonnet angle is continuously monitored and used for post-impact analysis, including the evaluation of the head injury criterion (HIC). Additionally, the system allows the detection of the bonnet position at the moment of head contact, providing further insight into the effectiveness of the active bonnet deployment.

**Figure 4:**
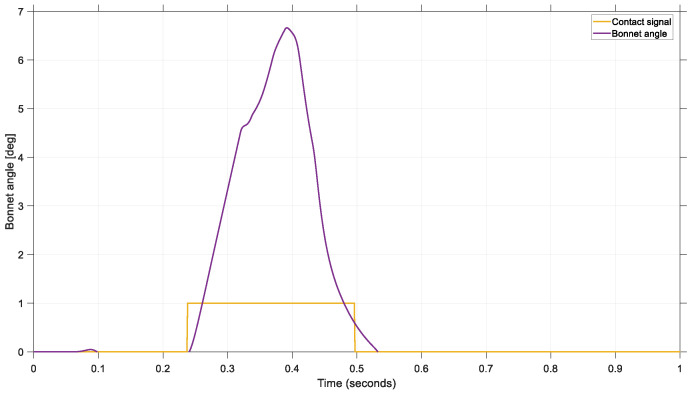
Time evolution of the contact signal and bonnet angle during the pedestrian–vehicle impact, highlighting the activation and response of the active bonnet system.

The dynamic response of the active bonnet system, including the contact signal and the resulting bonnet rotation, is presented in Figure 4.

As shown in Figure 4, the actuator is triggered immediately after the detection of the contact signal, resulting in a rapid increase in the bonnet angle. The maximum rotation is reached shortly after the activation phase, followed by a gradual decrease as the contact condition ceases and the system returns toward its equilibrium state. This behaviour confirms the effectiveness of the implemented control strategy in ensuring timely deployment of the active bonnet.

## 3. Model Parameters

The accuracy of the multibody simulation depends strongly on the proper definition of the physical and geometric parameters of both the pedestrian and the vehicle subsystems. In this study, a simplified yet biomechanically representative pedestrian model was implemented using rigid bodies interconnected through joints, with properties derived from anthropometric data and adjusted for numerical stability. The pedestrian body was discretized into multiple segments, including the head, torso, pelvis, upper and lower limbs. Each segment was assigned a specific mass, geometry, and inertia tensor, as summarized in Table 2.

The geometric dimensions of each segment are defined according to the simplified shapes used in the multibody model. For prismatic bodies such as the torso and pelvis, the dimensions are expressed as length, width, and height [L, W, H]. For cylindrical segments representing the limbs, the dimensions are given as radius and length [R, L].

The anthropomorphic dummy used for experimental validation was a custom-built research dummy designed to reproduce the global kinematic behaviour of a pedestrian during vehicle impact. The segment masses and inertial properties used in the numerical model are summarized in Table 2 and were selected to reproduce the main mass distribution of the physical dummy. However, the dummy is not a certified Euro NCAP or NHTSA pedestrian impactor and does not reproduce the full biofidelity of standardized human body models or finite element pedestrian models. Consequently, the validation presented in this study should be interpreted as a model-to-test correlation for global pedestrian kinematics and head acceleration response, rather than as a regulatory certification procedure. The model is therefore intended for comparative evaluation of active bonnet deployment strategies, not for detailed prediction of all anatomical injury mechanisms.

The moments of inertia are defined with respect to the local coordinate system of each segment, centred at the centre of mass. These parameters were introduced directly in the Simscape Multibody environment to ensure consistency between the geometric representation and the dynamic response.

The dynamic behaviour of the pedestrian model is further governed by the joint definitions connecting the body segments. Each joint was assigned a specific type, along with stiffness and damping coefficients, to approximate the biomechanical response of the human body during impact. The joint configuration and associated parameters are summarized in Table 3.

The pedestrian model was implemented as a rigid multibody system to maintain computational efficiency and enable multiple simulation scenarios. Therefore, local deformation effects and detailed soft-tissue behaviour are not explicitly represented. This simplification may influence the accuracy of torso response, while the head kinematics and acceleration—used for injury assessment—remain well captured at a global level.

The joint types were selected based on the anatomical characteristics of each articulation. Spherical joints were used to allow multi-directional rotation in regions such as the shoulders, elbows, and torso connections, while revolute joints were employed for joints with predominant single-axis motion, such as the knees, ankles, and head–neck interface. The stiffness and damping coefficients were introduced to model the passive resistance of the joints and to ensure numerical stability during simulation. Higher stiffness values were assigned to critical joints involved in impact load transmission, such as the knees and ankles, while lower or zero stiffness values were used in upper body joints to allow more natural motion. For certain joints, such as the shoulders and elbows, zero stiffness values were considered to allow free rotational motion and to simplify the model without significantly affecting the global kinematics of the pedestrian. In contrast, joints in the lower limbs were assigned non-zero stiffness and damping values to better capture load transfer during ground contact and impact with the vehicle.

The interaction between the pedestrian and the vehicle, as well as with the ground, was modelled using nonlinear contact elements based on a smooth spring–damper formulation. This approach allows continuous force generation as a function of penetration depth and relative velocity, avoiding numerical instabilities associated with rigid contact formulations.

The contact parameters (Table 4) were defined for multiple interaction pairs, including the lower limbs with the vehicle bumper, the torso and head with the bonnet, and the overall body with the ground. Each contact pair was assigned specific stiffness and damping coefficients to reproduce the energy dissipation and deformation characteristics observed during pedestrian impacts.

It should be noted that the contact parameters used in this study represent equivalent interaction properties rather than exact material characteristics. The simplified spring–damper formulation was selected to ensure numerical stability and to achieve agreement with experimental acceleration signals. While more advanced nonlinear or finite element-based models can capture detailed material behaviour, the present approach is suitable for system-level analysis of pedestrian kinematics and injury metrics.

A larger transition region was used for the head–bonnet contact to better capture the distributed deformation and compliant behaviour of the bonnet surface, especially in the presence of the active bonnet mechanism. A constant friction coefficient of 0.3 was assumed for all contact pairs, representing typical interaction conditions between clothing, human skin, and vehicle surfaces. The higher damping value used for the pedestrian–ground interaction accounts for the energy dissipation during post-impact contact, reducing rebound effects and stabilizing the simulation. The adopted contact model ensures a stable and physically consistent representation of the impact events, enabling accurate prediction of pedestrian kinematics and injury-related metrics. In addition to the pedestrian and contact model parameters, the simulation setup and active bonnet system characteristics were defined to reproduce the dynamic response of the vehicle–pedestrian interaction. The same friction coefficient was assigned to all contact pairs to maintain numerical stability and reduce the number of uncertain parameters in the model. This assumption represents a simplification, since the real interaction between clothing, footwear, skin, asphalt, painted bumper surfaces, and metallic bonnet panels may involve different frictional behaviour. Therefore, the adopted value should be considered an equivalent friction parameter used for system-level comparison rather than an exact physical property for each contact interface. Future work will investigate pair-specific friction coefficients and their influence on pedestrian rotation, sliding behaviour, and head impact location. The main simulation parameters, along with the actuator and bonnet properties, are summarized in Table 5.

The vehicle impact speed was set to 17 km/h, corresponding to a low-speed collision scenario typically associated with urban environments. The simulation time and step size were selected to ensure sufficient temporal resolution, particularly for the accurate evaluation of injury criteria such as the Head Injury Criterion (HIC). A fixed-step solver (ODE3) with a time step of 0.001 s was used to ensure numerical stability and to capture the rapid dynamics of the impact event. This time resolution was also selected to improve the accuracy of discrete signal processing, particularly in the computation of HIC values. The active bonnet mechanism was modelled as a torque actuator applied in a revolute joint representing the bonnet hinge. The actuator torque was set to 1000 N·m, providing a rapid rotation of the bonnet upon activation. The hinge stiffness and damping coefficients were introduced to simulate the mechanical resistance of the bonnet system and to control its dynamic response. These parameters influence both the maximum bonnet angle and the post-impact behaviour. The maximum bonnet angle reached during the simulation was approximately 6.7 degrees, indicating a moderate lifting of the bonnet intended to reduce head injury severity. The activation of the bonnet system was controlled by a contact-based trigger signal, ensuring that the actuator is engaged only after the detection of pedestrian–vehicle interaction. A deployment delay of 0.15 s was introduced to simulate realistic actuator response times observed in active safety systems. The head injury criterion was evaluated using a 15 ms time window (HIC15), in accordance with commonly accepted standards for pedestrian safety assessment.

These parameters collectively define the simulation framework and ensure a consistent representation of both mechanical and control aspects of the active bonnet system within the multibody model.

## 4. Model Validation

The two experimental velocities were selected based on the available controlled impact tests and their relevance for low-speed urban pedestrian collisions. The 17 km/h test represents a low-speed impact condition suitable for initial model calibration and validation, while the 29 km/h test represents a more severe urban impact case close to the transition range in which the pedestrian head trajectory and active bonnet effectiveness become more sensitive to impact configuration. These two tests were therefore used to validate the model at two distinct impact severities, while the additional simulations at other velocities were treated as predictive comparative investigations rather than direct experimental validation cases.

To ensure the reliability of the developed multibody model, a validation process was performed by comparing the simulation results with experimental data obtained from controlled pedestrian impact tests. The validation focuses on key biomechanical indicators, including head and torso resultant accelerations, which are critical for injury assessment.

The experimental validation was conducted using a custom-built anthropomorphic dummy instrumented with a tri-axial accelerometer mounted at the head level and a PIC DAQ MT5 acquisition system (DSD Dr. Steffan Datentechnik Ges.m.b.H, Linz, Austria) positioned in the torso region. The head acceleration was recorded using a high-frequency tri-axial accelerometer with a sampling rate of up to 10 kHz per channel, while the torso signals were acquired through the PIC DAQ system operating at 1 kHz per channel.

The test setup also included a high-speed camera (FAST TECH HISPEC 5) (Fastec Imaging Corporation, San Diego, CA, USA) to capture the impact kinematics, enabling a direct comparison between the simulated and experimental motion of the pedestrian during the collision event. It should be noted that the initial validation was performed at 17 km/h, while an additional validation case was subsequently introduced at approximately 29 km/h. Therefore, the simulations at the remaining velocities are used for comparative analysis of system behaviour rather than direct experimental validation.

**Figure 5:**
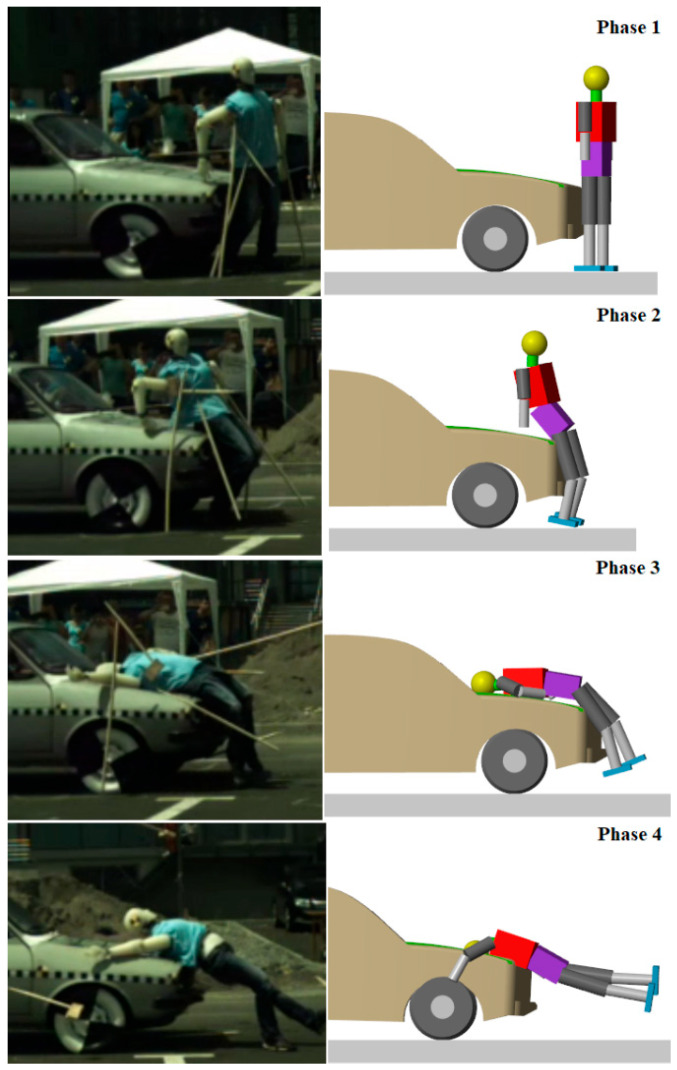
Kinematic validation of the 17 km/h pedestrian impact test: (**left**) high-speed camera frames from the experimental test; (**right**) corresponding simulation snapshots illustrating the main impact phases.

A qualitative comparison between the experimental test and the simulated pedestrian kinematics is presented in Figure 5, highlighting the main phases of the impact event.

As shown in Figure 5, the simulation accurately reproduces the main phases of the pedestrian–vehicle impact. In Phase 1, the initial contact occurs between the lower limbs and the vehicle front. In Phase 2, the torso rotates forward due to the impact forces, while the lower limbs remain in contact with the vehicle. Phase 3 corresponds to the head and upper body impact with the bonnet, representing the most critical moment in terms of injury risk. Finally, in Phase 4, the pedestrian body continues its forward motion, sliding over the vehicle surface. The good agreement observed between the experimental frames and the simulated configurations confirms that the model captures the essential kinematic behaviour of the pedestrian during the impact.

Following the qualitative validation of the kinematic response, a quantitative assessment was performed to evaluate the agreement between the simulated and experimental signals. The comparison was carried out using widely accepted statistical indicators, including the Root Mean Square Error (RMSE), Mean Absolute Error (MAE), and Peak Error, which are commonly employed in biomechanical modelling and impact analysis to quantify the accuracy of numerical simulations relative to experimental measurements [31,32,33]. The Root Mean Square Error (RMSE), defined in Equation (11), represents a global measure of the deviation between the simulated and experimental signals. It provides a weighted indication of large errors due to the squaring operation and is particularly sensitive to peak discrepancies, which are critical in impact dynamics and injury assessment. The Mean Absolute Error (MAE), given in Equation (12), evaluates the average magnitude of the absolute differences between the two signals, without emphasizing large deviations. This metric offers a more balanced representation of the overall error and is less influenced by outliers compared to RMSE [31]. The Peak Error, defined in Equation (13), quantifies the relative difference between the maximum values of the simulated and experimental signals. This parameter is especially important in crash analysis, as peak accelerations are directly correlated with injury risk and are often used in safety regulations and standards [32]. In addition to these statistical indicators, the Head Injury Criterion (HIC), previously defined in Equation (7), was used to evaluate the severity of head impact. The HIC combines both the magnitude and duration of head acceleration into a single metric and is widely adopted in automotive safety standards and pedestrian protection studies [34,35,36]. Pedestrian injury tolerance and vehicle–pedestrian multibody impact responses have also been investigated in previous studies [37,38].

The HIC value is calculated over a time interval [*t*_1_, *t*_2_], typically limited to 15 ms (HIC15), and is strongly correlated with the likelihood of head injury. In the above formulations, *y_i_^sim^* and *y_i_^test^* represent the simulated and experimental signal values at the i-th time step, *n* is the total number of samples, and *y_max_* denotes the peak value of the respective signals. The acceleration *a*(*t*) used in the HIC computation corresponds to the resultant head acceleration expressed in units of gravitational acceleration (g).

(11) $$RMSE=\sqrt{\frac{1}{n}\sum_{i=1}^{n}{\left(y_{i}^{sim}-y_{i}^{test}\right)}^{2}}$$

(12) $$MAE=\frac{1}{n}\sum_{i=1}^{n}\left|y_{i}^{sim}-y_{i}^{test}\right|$$

(13) $$Peak\;Error=\frac{|y_{max}-y_{max}|}{y_{max}}\times 100$$

These indicators provide complementary information regarding the accuracy of the model, capturing both absolute and relative differences, as well as peak response discrepancies and injury-related metrics. The HIC was computed using a discrete implementation with a fixed time step, ensuring consistency with the sampling rate of the experimental data.

**Figure 6:**
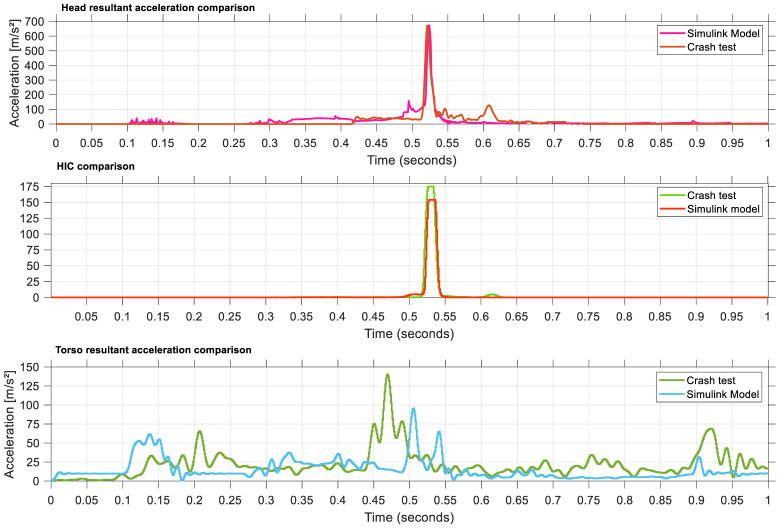
Comparison between simulated and experimental results: head resultant acceleration, HIC15 evolution, and torso resultant acceleration.

The validation of the multibody model was performed by comparing the simulated and experimental signals for head and torso resultant accelerations, as well as the corresponding Head Injury Criterion (HIC). The results are presented in Figure 6.

As shown in Figure 6, the simulated head acceleration closely follows the experimental signal, particularly in the main impact region around 0.5 s, where the peak value is accurately reproduced. The HIC evolution exhibits a similar trend, with both signals reaching their maximum values within the same time interval, indicating a good temporal and amplitude correlation. For the torso acceleration, a reasonable agreement is observed, although some discrepancies appear in the secondary peaks and post-impact phase. These differences may be attributed to simplifications in joint modelling and contact parameterization. The quantitative validation results are summarized in Table 6.

The statistical indicators confirm the validity of the proposed model. For the head acceleration, an RMSE value of 31.20 m/s^2^ and a peak error below 1% indicate a very good agreement between simulation and experimental data. The torso acceleration presents higher deviations, with an RMSE of 23.33 m/s^2^ and a peak error of 31.74%, which can be considered acceptable given the complexity of torso dynamics and the multiple contact interactions involved.

Overall, the model demonstrates a satisfactory capability to reproduce the key kinematic and dynamic characteristics of pedestrian impact, supporting its use for further injury analysis and evaluation of active safety systems.

### 4.1. Additional Validation at 29 km/h

To further evaluate the predictive capability of the proposed multibody model beyond the initial 17 km/h validation case, an additional pedestrian impact test performed at approximately 29 km/h was reproduced numerically. The objective of this supplementary validation was to assess the capability of the model to reproduce pedestrian kinematics and head acceleration response in a higher-speed impact configuration, corresponding to the transition region where the effectiveness of the active bonnet system becomes more dependent on the impact geometry and pedestrian motion.

**Figure 7:**
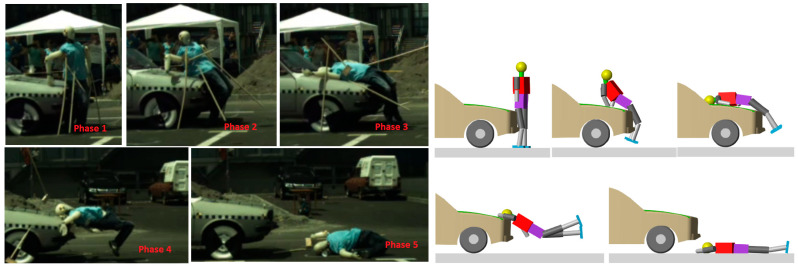
Comparison between experimental and simulated pedestrian kinematics during the impact: (**left**) high-speed camera frames from the experimental test; (**right**) corresponding simulation snapshots illustrating the main impact phases at 29 km/h.

Figure 7 presents the frame-by-frame comparison between the experimental pedestrian impact test performed at approximately 29 km/h and the corresponding multibody simulation. The comparison demonstrates that the proposed model reproduces the main kinematic phases of the impact with satisfactory agreement. In both the experimental test and the numerical simulation, the initial contact occurs at the lower limbs, followed by torso rotation toward the hood surface. Subsequently, the upper body and head interact with the hood region, while the lower limbs continue rotating due to the vehicle-induced angular momentum. The final post-impact body motion and ground contact sequence are also reproduced consistently by the model.

The comparison further shows that the simulated head trajectory and torso motion remain close to the experimental observations throughout the impact sequence, supporting the capability of the multibody model to capture the dominant pedestrian impact dynamics. In particular, the simulation correctly reproduces the forward torso rotation, the head impact location on the hood, and the overall body orientation during the post-impact phase.

A local discrepancy can be observed in the lower-limb motion, particularly at the knee joints during the later impact stages. In the experimental test, the legs remain closer to the ground surface, whereas in the simulation the lower limbs are lifted more noticeably upward. This difference may be attributed to the simplified representation of the lower-limb biomechanics, the absence of clothing and footwear interaction effects, and the idealized ground contact conditions used in the multibody model. In the physical dummy, the jeans, shoes, joint friction, and mechanical joint limits likely introduced additional resistance and energy dissipation, reducing the upward rotation of the legs after the primary body impact.

Nevertheless, this discrepancy mainly affects the secondary lower-limb posture during the post-impact phase and does not significantly influence the head trajectory, torso kinematics, or the primary head impact characteristics, which represent the main quantities of interest in the present study.

Besides the frame-by-frame kinematic validation, the resultant head acceleration was also compared in order to evaluate the capability of the model to reproduce the main dynamic characteristics of the pedestrian impact.

**Figure 8:**
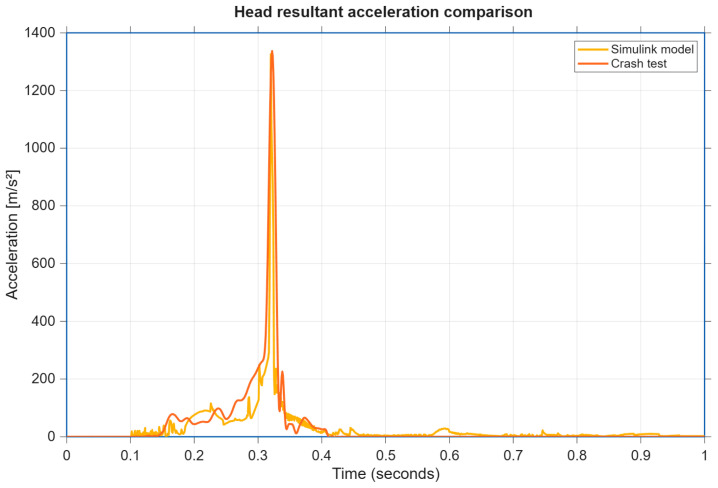
Comparison between simulated and experimental results: head resultant acceleration.

Figure 8 presents the comparison between the simulated and experimentally measured resultant head acceleration for the additional 29 km/h validation case. The comparison shows good agreement regarding the timing, amplitude, and overall shape of the primary impact pulse. The model reproduces the dominant acceleration peak associated with the head-to-hood impact, confirming the capability of the proposed multibody framework to capture the main dynamic characteristics of the pedestrian collision.

Although the peak acceleration values are closely matched, some differences remain in the pulse width and post-impact oscillations. The experimental signal exhibits a slightly broader acceleration pulse, resulting in a longer effective impact duration. This behaviour may be attributed to local structural compliance, simplified contact damping, and the absence of soft-tissue deformation effects in the multibody model. Nevertheless, the agreement between simulation and experiment remains satisfactory for the intended head injury assessment applications.

To further quantify the agreement between the simulated and experimental head acceleration signals, several statistical validation indicators were calculated, including the Root Mean Square Error (RMSE), Mean Absolute Error (MAE), and Peak Error. The obtained validation metrics are summarized in Table 7.

The obtained statistical indicators confirm a satisfactory agreement between the numerical model and the experimental measurements. In particular, the very low peak error demonstrates the capability of the proposed model to accurately reproduce the dominant head impact pulse, while the RMSE and MAE values remain acceptable considering the highly transient and nonlinear nature of pedestrian impact dynamics.

### 4.2. Parametric Sensitivity Analysis

To further evaluate the influence of the sensing and actuation parameters on pedestrian head injury mitigation, a parametric sensitivity analysis was performed using the validated multibody model. The analysis focused on two key parameters of the active bonnet system: the actuator deployment delay and the actuator torque. These parameters directly influence the bonnet lifting dynamics and the available deformation space prior to head contact.

#### 4.2.1. Deployment Delay Influence

The deployment delay $t_{d}$ represents the time interval between the detection of pedestrian contact and the activation of the bonnet actuator. Three representative delay values were investigated: 0 s (baseline instantaneous activation), 0.10 s, and 0.15 s. The corresponding peak head acceleration and HIC15 values are summarized in Table 8.

The results indicate that the effectiveness of the active bonnet system decreases as the deployment delay increases. Earlier activation allows the bonnet to reach a larger angular displacement before head impact occurs, increasing the available deformation space and reducing the severity of the collision. In contrast, delayed deployment reduces the bonnet lift available at the moment of head contact, leading to increased peak acceleration levels and higher HIC15 values. For the baseline configuration without additional delay, the model produced a peak head acceleration of 91 m/s^2^ and an HIC15 value of 181. When the delay increased to 0.15 s, the peak acceleration increased to 102 m/s^2^, while the HIC15 value increased to 245. These results emphasize the importance of rapid impact detection and fast actuator response for effective pedestrian protection.

#### 4.2.2. Actuator Torque Influence

The actuator torque $T_{act}$ controls the angular acceleration and final lift position of the bonnet during deployment. Three representative torque values were analysed: 500 N·m, 1000 N·m (baseline), and 1500 N·m. The obtained results are presented in Table 9.

The analysis shows that the actuator torque strongly influences the bonnet deployment dynamics and the resulting injury metrics. Lower torque values generate a slower bonnet lifting motion, reducing the deformation space available during head impact and increasing the injury severity. Conversely, higher actuator torques accelerate the bonnet deployment process and improve the energy absorption capability of the system. For the reduced torque configuration of 500 N·m, the model generated a peak head acceleration of 84 m/s^2^ and an HIC15 value of 264. Increasing the torque to 1500 N·m reduced the peak acceleration to 76 m/s^2^ and maintained the HIC15 value close to the baseline configuration. These findings indicate that actuator torque represents a critical design parameter influencing the effectiveness of active pedestrian protection systems.

Although the reduced torque configuration generated a slightly lower peak acceleration compared to the baseline case, the corresponding acceleration pulse exhibited a longer effective duration, resulting in an increased HIC15 value.

#### 4.2.3. Discussion of Sensitivity Results

Overall, the sensitivity analysis demonstrates that the performance of the active bonnet system is strongly dependent on the synchronization between pedestrian detection and bonnet deployment. Both excessive deployment delays and insufficient actuator torque can significantly reduce the protective capability of the system, particularly in impact scenarios where the available deployment time is limited. The obtained results highlight the importance of optimizing both the sensing and actuation subsystems in real-world active bonnet implementations. Although the present study employs a simplified actuator representation, the parametric trends remain consistent with the expected physical behaviour of deployable pedestrian protection systems and provide useful insight into the influence of response timing and deployment dynamics on head injury mitigation.

Although the actuator model used in this study is simplified and does not reproduce the detailed behaviour of pyrotechnic deployment systems, the obtained trends remain representative for comparative evaluation purposes.

## 5. Results and Discussion

### 5.1. Comparative Results

To evaluate the effectiveness of the active bonnet system, a series of simulations were performed at different vehicle impact velocities: 17, 20, 25, 28, 30, and 40 km/h. This comparative approach allows assessing the influence of the active system on pedestrian head injury metrics and impact dynamics.

**Figure 9:**
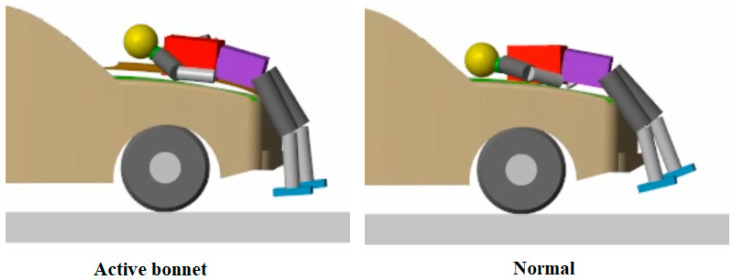
Comparison of pedestrian head impact for velocities up to 30 km/h: (**left**) active bonnet configuration; (**right**) reference configuration.

For each scenario, two configurations were analysed: a reference case (without active bonnet) and an active bonnet case. A qualitative comparison of the pedestrian kinematics for impact velocities up to 30 km/h is presented in Figure 9.

**Figure 10:**
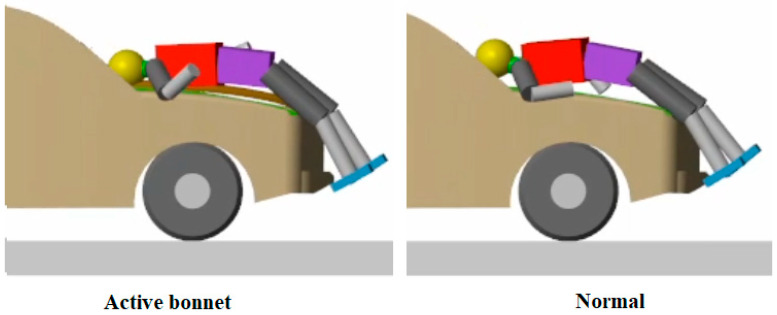
Comparison of pedestrian head impact at higher velocities (30–40 km/h): (**left**) active bonnet configuration; (**right**) reference configuration.

As shown in Figure 9, for impact velocities up to 30 km/h, the active bonnet system increases the distance between the head and the rigid components of the engine compartment. This results in a softer contact surface and a more favourable distribution of impact forces. In contrast, in the reference configuration, the head impacts a stiffer structure, leading to higher deceleration levels. The kinematic behaviour at higher impact velocities (30–40 km/h) is illustrated in Figure 10.

At higher velocities, the effectiveness of the active bonnet system decreases significantly. As illustrated in Figure 10, the head impact shifts from the bonnet surface to the windshield area. In this case, the lifting of the bonnet no longer provides sufficient protection, as the contact occurs with a much stiffer structure, resulting in higher acceleration peaks and increased HIC values. These observations explain the trends observed in the acceleration and HIC results presented in the following sections.

**Figure 11:**
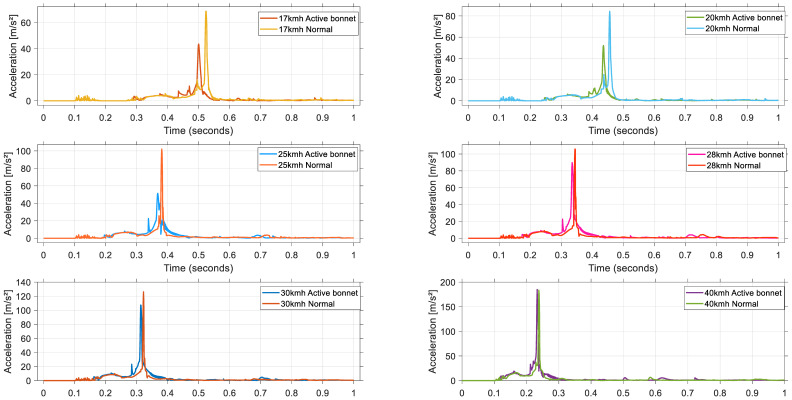
Comparison of head resultant acceleration for different impact velocities, with and without active bonnet.

**Figure 12:**
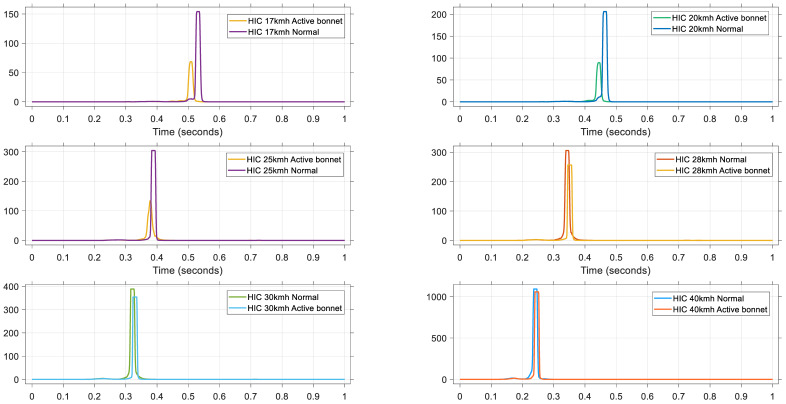
Comparison of HIC15 values for different impact velocities, with and without active bonnet.

As shown in Figure 11, the active bonnet system significantly reduces the peak head acceleration at lower impact velocities. For velocities of 17 km/h and 20 km/h, a substantial decrease in peak acceleration is observed, indicating effective energy absorption due to bonnet lifting. At intermediate velocities (25–30 km/h), the reduction becomes less pronounced, suggesting a limited effectiveness of the system. At higher velocities (40 km/h), the difference between the two configurations becomes negligible, indicating that the active bonnet is no longer able to sufficiently mitigate the impact severity. The corresponding HIC15 evolution for the same scenarios is illustrated in Figure 12.

The HIC results confirm the trends observed in the acceleration signals. At lower velocities (17–25 km/h), the active bonnet leads to a significant reduction in HIC values, with decreases exceeding 50% in some cases. This highlights the effectiveness of the system in reducing head injury risk in typical urban collision scenarios. However, at higher velocities (above 28 km/h), the effectiveness decreases, and in some cases, the HIC values obtained with the active bonnet exceed those of the reference configuration. This behaviour can be attributed to insufficient bonnet displacement and reduced time available for energy absorption, limiting the protective effect of the system. The quantitative comparison of peak head accelerations is summarized in Table 10.

The results indicate a substantial reduction in peak head acceleration for lower velocities, with decreases of up to approximately 60% at 17 and 20 km/h. As the velocity increases, the reduction becomes less significant, and at 40 km/h, the active bonnet does not provide a measurable improvement. The bonnet angle at head contact also decreases with increasing velocity, indicating a reduced deployment effectiveness at higher impact speeds.

In addition to RMSE, MAE, and Peak Error, the Pearson correlation coefficient was calculated to further assess the similarity between the simulated and experimental signals. A Pearson correlation coefficient of R = 0.85 was obtained for the head acceleration signal. This value was used only as a supplementary indicator of signal similarity, together with RMSE, MAE, Peak Error, and kinematic comparison, rather than as the primary validation criterion. The corresponding HIC values for all simulated scenarios are presented in Table 11.

At 17 km/h and 20 km/h, the active bonnet system provides a clear reduction in both peak head acceleration and HIC. However, at 30 km/h and 40 km/h, the benefit decreases substantially. The kinematic analysis indicates that, at these higher speeds, the pedestrian head trajectory extends toward the windshield, so the impact no longer occurs mainly on the bonnet. Consequently, the active bonnet has little protective effect, since its action is limited to modifying the bonnet-head contact conditions and cannot significantly mitigate impacts occurring in the windshield region.

Additionally, the two experimental validation tests were performed using different pedestrian impact configurations relative to the vehicle front-end geometry. In the initial 17 km/h validation case, the pedestrian trajectory was more offset relative to the longitudinal axis of the vehicle, resulting in a head trajectory directed closer toward the windshield region during the later stages of the impact. In contrast, the additional 29 km/h validation test involved a more central pedestrian position relative to the hood surface, producing a different body rotation mechanism and head impact location.

**Figure 13:**
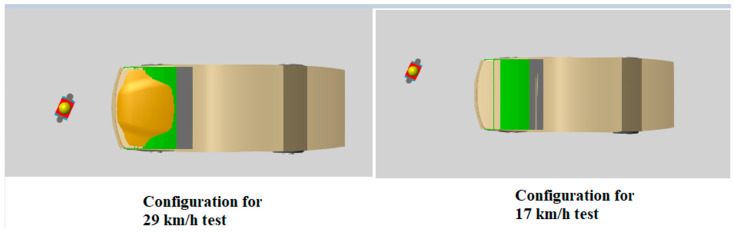
Pedestrian impact configurations used in the study.

Since pedestrian impact configuration strongly influences the subsequent head trajectory and the available deformation space prior to head contact, additional numerical simulations were performed using the pedestrian positioning corresponding to the 29 km/h experimental test. The objective of these supplementary simulations was to evaluate the influence of pedestrian orientation and impact location on the effectiveness of the active bonnet system across different vehicle velocities. Figure 13 illustrates the different pedestrian impact configurations used in the study. In the 29 km/h validation test, the pedestrian impact occurred closer to the central hood region, whereas the 17 km/h configuration involved a more offset trajectory relative to the vehicle longitudinal axis.

The comparison shown in Figure 13 highlights the geometric differences between the two pedestrian impact configurations. In the 29 km/h validation case, the pedestrian impact occurs closer to the central hood region, allowing the head to remain within the deformable bonnet area for a longer duration. Consequently, the active bonnet system retains a higher protective capability even at intermediate impact speeds. Conversely, in the more offset configuration associated with the 17 km/h validation scenario, the head trajectory tends to migrate more rapidly toward the windshield region as the vehicle speed increases, reducing the effectiveness of bonnet deployment.

The quantitative results obtained for the additional simulation campaign based on the 29 km/h pedestrian configuration are summarized in Table 12 and Table 13. The presented results include the comparison between the active bonnet and reference configurations in terms of peak head acceleration and HIC15 values for multiple vehicle velocities.

Overall, the additional simulation campaign demonstrates that the effectiveness of the active bonnet system is strongly influenced not only by vehicle velocity, but also by pedestrian impact configuration and head trajectory relative to the vehicle front-end geometry. For the pedestrian positioning corresponding to the 29 km/h validation test, the active bonnet maintained a significant protective effect across the investigated velocity range, reducing both the peak head acceleration and the corresponding HIC15 values compared with the reference configuration.

The results further indicate that more central pedestrian impact configurations allow the head to remain within the deformable bonnet region for a longer duration, increasing the available deformation space prior to head contact. This behaviour improves the capability of the active bonnet system to mitigate head injury severity, particularly at intermediate impact speeds. Nevertheless, at higher velocities such as 40 km/h, the reduction in injury metrics becomes less pronounced due to the increased kinetic energy and reduced available deployment time before head impact.

The approximately 15% HIC15 reduction observed at 40 km/h should be interpreted cautiously. Although it indicates that the active bonnet still modifies the head impact conditions, the magnitude of the reduction is limited compared with the reductions obtained at lower and intermediate velocities. Therefore, the active bonnet system alone may not be sufficient to ensure robust pedestrian head protection in higher-speed impacts. In such cases, additional safety strategies, such as optimized windshield base design, adaptive deployment control, or integration with pre-impact braking systems, may be required.

These findings confirm that pedestrian orientation, impact location, and deployment timing represent critical parameters in the design and optimization of active bonnet systems and should be considered together with vehicle speed when evaluating pedestrian protection performance.

### 5.2. Discussion

The results obtained in the previous section highlight both the strengths and limitations of the active bonnet system in mitigating pedestrian head injury. The observed reduction in peak acceleration and HIC values at lower impact velocities can be explained by the additional deformation space created between the bonnet and the underlying rigid components of the engine compartment. At lower velocities, the active bonnet has sufficient time to deploy before the head impact occurs, allowing the system to absorb part of the impact energy through controlled deformation and increased stopping distance. This results in a lower deceleration rate of the head, which directly contributes to reduced HIC values. From a dynamic perspective, the effectiveness of the system is governed by the relationship between impact velocity, actuator response time, and bonnet displacement. As the vehicle speed increases, the time available for bonnet deployment decreases, leading to a reduced lift angle at the moment of head contact.

Although HIC15 values were calculated according to the standard formulation, they should be interpreted primarily as comparative indicators between the investigated configurations rather than as exact clinical injury predictions. The multibody model captures the global pedestrian kinematics and resultant head acceleration response, but it does not include detailed local deformation of the head, soft tissues, bonnet structure, or windshield components. Consequently, the HIC15 results are suitable for evaluating relative trends between reference and active bonnet configurations, while more detailed injury prediction would require finite element or highly calibrated compliant body models.

#### 5.2.1. Transition of Head Impact Location at Higher Velocities

At higher vehicle velocities, the pedestrian kinematics become increasingly sensitive to the initial impact configuration, pedestrian orientation, and head trajectory relative to the vehicle front-end geometry. The initial simulation campaign, based on the 17 km/h validation configuration, indicated a progressive reduction in active bonnet effectiveness above approximately 28–30 km/h. In this configuration, the head trajectory shifted toward the windshield region as the impact speed increased, reducing the available deformation space provided by the deployable bonnet system.

It should also be noted that the present study was performed using a single simplified passenger-car front-end geometry. Parameters such as bumper height, bonnet leading-edge height, bonnet length, and windshield angle strongly influence pedestrian rotation and head impact location. Therefore, the trends reported in this study should not be generalized directly to other vehicle classes, such as SUVs, vans, or vehicles with substantially different front-end geometries, without additional calibration and validation. Future work will extend the proposed framework to multiple vehicle front-end profiles to evaluate the robustness of active bonnet deployment strategies across different vehicle categories.

However, the additional simulations performed using the pedestrian positioning corresponding to the 29 km/h experimental validation test revealed a different behaviour. In the more central impact configuration, the pedestrian head remained within the deformable hood region for a longer duration, allowing the active bonnet system to maintain a significant protective effect even at intermediate impact velocities. The obtained results demonstrated substantial reductions in both peak head acceleration and HIC15 values compared with the reference configuration.

These observations indicate that the effectiveness of active bonnet systems cannot be evaluated solely as a function of vehicle velocity. Instead, the injury mitigation capability strongly depends on the combined influence of impact speed, pedestrian positioning, head impact location, body rotation mechanism, and the available deployment time before head contact. Pedestrian trajectories directed toward the windshield base or stiffer structural regions tend to reduce the protective capability of bonnet deployment systems, whereas impacts concentrated within the central hood region allow more efficient energy dissipation.

The results further highlight the importance of considering multiple pedestrian impact configurations during the design and validation of active pedestrian protection systems. The presented findings suggest that future active bonnet strategies may benefit from adaptive deployment algorithms capable of accounting not only for impact detection timing, but also for pedestrian trajectory prediction and probable head impact location.

Although the proposed multibody model includes several simplifications, including rigid body assumptions and simplified contact definitions, the obtained trends remain physically consistent with the experimental observations and demonstrate the capability of the proposed framework to evaluate the influence of impact configuration on pedestrian head injury mitigation.

#### 5.2.2. Practical Implementation of the Contact-Triggered Sensor

In the proposed numerical framework, the bonnet deployment is initiated using a contact-triggered signal generated when the pedestrian lower limbs interact with the vehicle bumper region. Although the present study uses an idealized trigger condition, the implemented logic was intended to reproduce the functional behaviour of real contact-based pedestrian detection systems used in active bonnet applications.

In practical vehicle applications, such a sensing mechanism could be implemented using pressure-tube sensors, accelerometer-based bumper sensors, or distributed deformation-sensitive elements integrated within the front bumper structure. Among these solutions, pressure-tube systems are commonly used in commercial pedestrian protection systems due to their rapid response time and relatively simple integration within the bumper assembly.

In a pressure-based implementation, the impact between the pedestrian leg and the bumper produces a localized pressure wave inside a deformable tube positioned behind the bumper fascia. The resulting pressure variation is detected by a sensor unit and processed by the vehicle control module, which subsequently activates the bonnet lifting actuator if predefined triggering thresholds are exceeded.

For possible physical implementation, the contact-triggered signal may be generated using a pressure-tube sensor positioned behind the bumper fascia. The impact between the pedestrian lower limb and the bumper produces a rapid pressure rise inside the tube, which can be converted into an electrical signal and evaluated by the vehicle control unit. A practical implementation would require a calibrated detection threshold, signal filtering, and plausibility checks. The threshold should be high enough to reject low-energy non-relevant contacts, such as small road debris or parking contacts, but low enough to ensure timely detection of pedestrian lower-limb impacts. In addition, the trigger logic may combine pressure amplitude, pressure rise rate, and impact duration to reduce false-positive and false-negative activations.

In the present numerical model, this process is represented by an idealized binary contact signal. Therefore, sensor noise, threshold uncertainty, signal filtering, and object classification are not explicitly simulated. The simplified trigger was adopted to isolate the influence of deployment timing and bonnet actuation on head injury metrics. Nevertheless, the proposed framework can be extended by replacing the binary trigger with a threshold-based sensor model including response time, noise, and decision logic.

Nevertheless, the adopted approach provides a simplified but physically representative framework suitable for analysing the interaction between pedestrian impact detection and active bonnet deployment dynamics.

**Figure 14:**
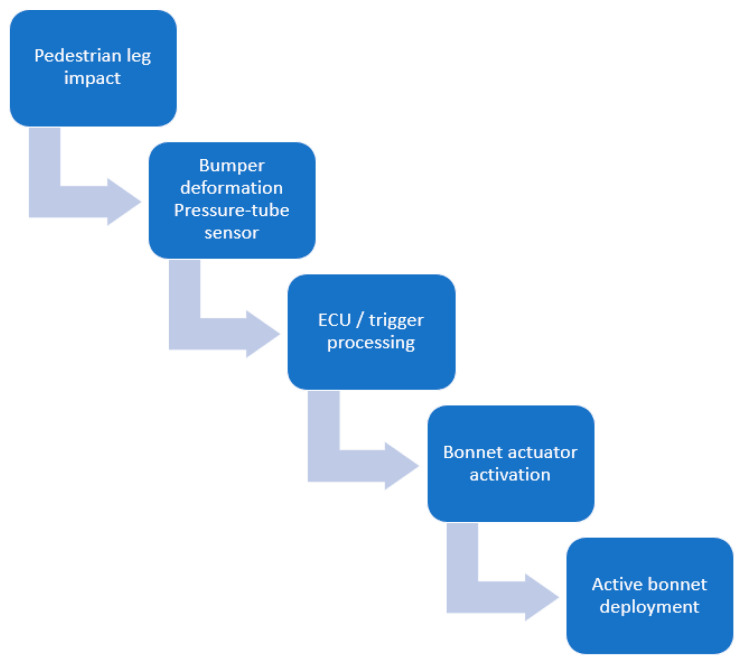
Conceptual implementation of the proposed contact-triggered active bonnet sensing and deployment system.

Figure 14 illustrates a conceptual implementation of the proposed contact-triggered sensing mechanism. In a practical vehicle application, the impact between the pedestrian lower limbs and the bumper structure generates a pressure variation detected by a pressure-tube sensor integrated within the bumper assembly. The resulting signal is processed by the vehicle ECU, which subsequently activates the bonnet deployment actuator.

Future developments may include detailed sensor modelling, signal-processing algorithms, threshold calibration, and discrimination strategies capable of distinguishing pedestrian impacts from non-relevant objects such as road debris or low-speed parking contacts.

## 6. Conclusions

This study proposed and evaluated a contact-triggered active bonnet approach for pedestrian head injury mitigation. The model was validated using controlled impact tests at approximately 17 km/h and 29 km/h, including kinematic comparisons and measured head acceleration signals. The validation results showed that the model can reproduce the dominant pedestrian motion and primary head impact response with satisfactory agreement.

The comparative simulations showed that active bonnet deployment can reduce peak head acceleration and HIC15 values, particularly when the pedestrian head remains within the deformable bonnet region. The results also demonstrated that system effectiveness is not governed by vehicle speed alone, but depends strongly on pedestrian positioning, head trajectory, and head impact location relative to vehicle front-end geometry.

The sensitivity analysis confirmed that deployment timing and actuator torque are critical design parameters. Increased deployment delay reduced the protective effect, while actuator torque influenced both the bonnet lift and the effective duration of the head acceleration pulse. These findings highlight the importance of coordinated sensing, decision logic, and actuator response in active pedestrian protection systems.

The proposed framework should be interpreted as a comparative design and evaluation tool rather than a regulatory injury prediction model. Future work will focus on detailed sensor modelling, nonlinear actuator representation, pair-specific contact and friction parameters, and validation using additional pedestrian dummy configurations and vehicle front-end geometries.

## Figures and Tables

**Table 1:** Simplified vehicle front-end geometry parameters.

Parameter	Value	Unit
Bumper height	507	mm
Bonnet leading edge height	733	mm
Bonnet angle	8	deg
Bonnet length	1170	mm
Windshield angle	38	deg

**Table 2:** Anthropometric and inertial properties of the pedestrian model segments.

Segment	Mass [kg]	Length [m]	Moment of Inertia [kg·m^2^]
Head	4.7	0.115	[0.024, 0.024, 0.024]
Torso	20	[0.25, 0.35, 0.3]	[0.35, 0.25, 0.30]
Pelvis	13	[0.2, 0.3, 0.2]	[0.14, 0.08, 0.14]
Left/Right upper arm	2.2	[0.06, 0.26]	[0.014, 0.014, 0.003]
Left/Right forearm	2.5	[0.045, 0.24]	[0.013, 0.013, 0.002]
Left/Right thigh	9.3	[0.07, 0.42]	[0.014, 0.014, 0.002]
Left/Right shank	4.3	[0.05, 0.38]	[0.05, 0.05, 0.005]
Left/Right foot	1.7	[0.25, 0.05, 0.1]	[0.001, 0.01, 0.009]

**Table 3:** Joint types used for pedestrian model.

Joint	Connected Segments	Joint Type	Stiffness	Damping
Head joint	Head–Neck	Revolute	0.05	0.02
Neck joint	Neck–Torso	Spherical	0.5	0.02
Right shoulder	Torso–Right upper arm	Spherical	0	0
Left shoulder	Torso–Left upper arm	Spherical	0	0
Right elbow	Right upper arm–Right forearm	Spherical	0	0
Left elbow	Left upper arm–Left forearm	Spherical	0	0
Lumbar/torso joint	Torso–Pelvis	Spherical	0	0
Right hip	Pelvis–Right thigh	Revolute	0	0
Left hip	Pelvis–Left thigh	Revolute	0	0
Right knee	Right thigh–Right shank	Revolute	0.5	0.05
Left knee	Left thigh–Left shank	Revolute	0.5	0.05
Right ankle	Right shank–Right foot	Revolute	0.5	0.05
Left ankle	Left shank–Left foot	Revolute	0.5	0.05

**Table 4:** Contact parameters.

Contact Pair	Contact Model	Stiffness [N/m]	Damping [N/(m/s)]	Transition Region Width [m]	Friction Coefficient [-]
Pedestrian lower limbs–Vehicle bumper	Smooth spring-damper	10,000	1000	1 × 10^−4^	0.3
Pedestrian torso–Hood	Smooth spring-damper	10,000	1000	1 × 10^−4^	0.3
Pedestrian head–Hood	Smooth spring-damper	10,000	1000	0.055	0.3
Pedestrian body–Ground	Smooth spring-damper	10,000	2000	1 × 10^−4^	0.3

**Table 5:** Simulation and active bonnet parameters.

Parameter	Symbol/Description	Value	Unit
Baseline vehicle impact speed	v	17	km/h
Simulation time	t_sim_	1	s
Solver type	ODE3	—	—
Simulation step size	delta_t_	0.001	s
Bonnet actuator type	Torque applied in revolute joint	—	—
Bonnet actuator torque	T_act_	1000	N·m
Hinge spring stiffness	k_h_	10	N·m/rad
Hinge damping coefficient	c_h_	15	N·m·s/rad
Equilibrium bonnet angle	theta_0_	0	deg
Maximum bonnet angle reached	theta_max_	6.7	deg
Trigger signal	Contact signal	—	—
Deployment delay	t_d_	0.15	s
HIC evaluation window	HIC15	15	ms

**Table 6:** Statistical validation metrics for head and torso acceleration signals.

Signal	RMSE (m/s^2^)	MAE (m/s^2^)	Peak Error (%)	Agreement Level
Head acceleration	31.20	14.90	0.399	Very good
Torso acceleration	23.33	15.83	31.74	Acceptable

**Table 7:** Statistical validation metrics for head acceleration signals.

Signal	RMSE (m/s^2^)	MAE (m/s^2^)	Peak Error (%)	Agreement Level
Head acceleration	56.63	16.92	0.776	Very good

**Table 8:** Deployment delay comparison.

Deployment Delay [s]	Peak Acceleration [m/s^2^]	HIC15
0 (baseline)	91	181
0.10	93	212
0.15	102	245

**Table 9:** Actuator torque comparison.

Actuator Torque [N·m]	Peak Acceleration [m/s^2^]	HIC15 [-]
500	84	264
1000 (baseline)	91	181
1500	76	184

**Table 10:** Peak head acceleration and bonnet angle results for the 17 km/h validation-based configuration.

Vehicle Velocity [km/h]	Peak Acceleration—Active Bonnet [m/s^2^]	Peak Acceleration—Reference [m/s^2^]	Difference [%]	Bonnet Angle at Head Contact [deg]
17 (validation)	43.49	68.66	−57.89	4.53
20	52.07	84.38	−62.05	4.37
25	51.42	102	−49.59	0.03
28	89.72	105.8	−17.9	0.03
30	107.4	126.1	−17.47	0.034
40	185	183.5	0.77	0.043

**Table 11:** HIC15 results for the 17 km/h validation-based configuration.

Vehicle Velocity [km/h]	HIC Max—Active Bonnet	HIC Max—Reference	Difference [%]
17 (validation)	68.35	154.1	−55.65
20	89.3	206.3	−56.71
25	133.4	304.4	−56.18
28	306	256.4	16.2
30	388.5	354.2	9.68
40	1092	1058	3.2

**Table 12:** Peak head acceleration results for the 29 km/h validation-based configuration.

Vehicle Velocity [km/h]	Peak Acceleration—Active Bonnet [m/s^2^]	Peak Acceleration—Reference [m/s^2^]	Difference [%]
17 (validation)	64.9	72.15	−10.05
20	56	78	−28.21
25	97	118.7	−18.28
28	70	136	−48.53
30	80.4	137.6	−42.57
40	151.8	182.4	−16.78

**Table 13:** HIC15 results for the 29 km/h validation-based configuration.

Vehicle Velocity [km/h]	HIC Max—Active Bonnet	HIC Max—Reference	Difference [%]
17 (validation)	95	196	−51.53
20	118	176.8	−33.26
25	179.2	399	−55.09
28	171	549	−68.85
30	242.7	646	−62.43
40	968.9	1141	−15.08

## Data Availability

The data presented in this study are available on request from the corresponding author. The data are not publicly available due to their use in ongoing research activities.

## Abbreviations

The following abbreviations are used in this manuscript:

HIC	Head Injury Criterion
RMSE	Root Mean Square Error
MAE	Mean Absolute Error
MAPE	Mean Absolute Percentage Error
COG	Center of Gravity
DOF	Degrees of Freedom
CAD	Computer-Aided Design
